# Modulating gene translational control through genome editing

**DOI:** 10.1093/nsr/nwy123

**Published:** 2018-11-21

**Authors:** Feng Zhang, Daniel F Voytas

**Affiliations:** 1 Department of Plant and Microbial Biology, University of Minnesota, USA; 2 Center for Precision Plant Genomics, University of Minnesota, USA; 3 Department of Genetics, Cell Biology, and Development, University of Minnesota, USA; 4 Center for Genome Engineering, University of Minnesota, USA

Recent advances in genome editing have led to a wide range of applications that are not limited to the manipulation of gene function. One highly desirable application is to regulate gene expression in a targeted fashion. While gene expression is regulated at multiple levels, most research has focused on the transcriptional regulation of target genes by altering regulatory sequences, repurposing designer endonucleases to create artificial transcription factors or directly editing mRNA transcripts [1]. The control of gene expression at the translational level represents another important mechanism that has not been tapped into. In a recent issue of *Nature Biotechnology*, Gao's group from the Chinese Academy of Sciences demonstrated a simple and potentially widely applicable approach to modulate translational control of gene expression by editing upstream open reading frames (uORFs), the short protein-coding sequences located in the 5′ leader region of primary open reading frames (pORFs) [2].

uORFs exist in a large fraction of eukaryotic genes; for example, 49% of human genes, 44% of mouse genes and more than 40% of genes in various plant species, including *Arabidopsis*, maize and rice. Many of these genes are involved in important biological processes, such as gene regulation, development, metabolite biosynthesis and disease resistance. Emerging evidence has suggested that the presence of uORFs correlates with significantly reduced protein expression levels, likely because they reduce the efficiency of translation initiation at pORFs [3,4]. Gao's group hypothesized that disruption of the uORF would increase the translation of downstream pORFs. Using clustered regularly interspaced short palindromic repeats/Cas technology, they introduced targeted mutations in the uORFs of four plant genes from *Arabidopsis* and lettuce. Remarkably, mutants with disrupted uORFs from all four tested genes displayed significant increases in the expression of the primary protein product. Higher expression levels also resulted in pronounced phenotypes. For example, mutating uORFs in the key enzyme for ascorbate (vitamin C) biosynthesis, the GDP-L-galactose phosphorylase (GGP) gene, increased vitamin C content by 150%. Increasing levels of vitamin C has been a long sought-after target for crop improvement. The mutant lettuce lines also showed an increase in tolerance to oxidative damage. Moreover, for each uORF that was edited, multiple independent mutant lines were obtained with varying levels of protein expression, thereby providing the opportunity to further fine-tune translational controls.

Despite their widespread presence in plants and animals, little conservation was found across uORFs, and most of their functions remain to be elucidated [5]. Targeted mutagenesis of uORFs through genome editing opens doors to dissect these regions, and better understand mechanisms of translational control and attenuation in eukaryotes. In addition, this method offers a simpler and more generalizable way to upregulate gene expression by creating small indel mutations in uORFs. In contrast, previous approaches have often involved large insertions of foreign DNA fragments, such as sequences of strong promoters, enhancers or engineered artificial transcription activators, in the genome. Transgene-free mutant lines can be readily obtained using uORF editing, which may offer an advantage in crop improvement due to less regulatory constraints. Without a doubt, the uORF targeting method promises to have many applications in both basic and applied research.
